# Increased Expression of *BIRC2*, *BIRC3*, and *BIRC5* from the IAP Family in Mesenchymal Stem Cells of the Umbilical Cord Wharton's Jelly (WJSC) in Younger Women Giving Birth Naturally

**DOI:** 10.1155/2020/9084730

**Published:** 2020-04-08

**Authors:** Paulina Gil-Kulik, Małgorzata Świstowska, Adrianna Kondracka, Piotr Chomik, Arkadiusz Krzyżanowski, Anna Kwaśniewska, Mansur Rahnama, Janusz Kocki

**Affiliations:** ^1^Department of Clinical Genetics, Medical University of Lublin, Lublin, Poland; ^2^Department of Obstetrics and Pathology of Pregnancy, Medical University of Lublin, Lublin, Poland; ^3^Chair and Department of Dental Surgery, Medical University of Lublin, Lublin, Poland

## Abstract

The knowledge of factors affecting the viability as well as proliferation and therapeutic potential of perinatal stem cells is of great importance for the decisions concerning their collection, multiplication, and storing. The aim of this work is to evaluate the expression of the *BIRC2*, *BIRC3*, and *BIRC5* genes at the level of transcription in mesenchymal stem cells derived from the umbilical cord Wharton's jelly. The study examined the relationship between the expression level of the studied genes and selected biophysical parameters of umbilical blood: pH, pCO_2_, pO_2_, and cHCO_3_. Moreover, the relationship between the pregnant age, the type of delivery (natural delivery or cesarean section), and the level of expression of the *BIRC2*, *BIRC3*, and *BIRC5* genes was assessed. The research was carried out on mesenchymal stem cells derived from the umbilical cord Wharton's jelly (WJSC) taken from 55 women immediately after delivery. Expression of the examined genes was assessed with the qPCR method using commercially available reagent kits. On the basis of the conducted research, it was demonstrated that WJSCs collected from younger women giving birth naturally, and in the acidic environment of the umbilical cord blood, are characterized by a higher expression of the *BIRC2*, *BIRC3*, and *BIRC5* genes. It was shown that the expression of the *BIRC2* and *BIRC3* genes in Wharton's jelly mesenchymal stem cells declines with the mother's age. Our research suggests that stem cells collected from younger women giving birth naturally can be more resistant to apoptosis and show a more stem cell-like character, which can increase their therapeutic potential and clinical utility, but this conclusion needs to be approved in the next studies.

## 1. Introduction

Perinatal tissues are a valuable source of mesenchymal, hematopoietic, and fetal stem cells. Perinatal stem cells are characterized by greater plasticity and greater proliferative potential; they have lower immunogenic properties [1–4]. Due to the ease of collection and no side effects for both the mother and the child, obtaining perinatal stem cells does not raise moral or ethical concerns. Unfortunately, the potential of fetal stem cells is not yet fully exploited mainly due to the lack of appropriate isolation methods and techniques of efficient cell proliferation [5], as well as the fact that the factors affecting the viability and proliferative and therapeutic potential of collected cells are not known at this point [6].

The influence of various factors on the umbilical cord blood quality has been repeatedly studied [7–18]. However, there are very few studies on the influence of factors related to childbirth on the quality and potential of mesenchymal cells derived from the umbilical cord Wharton's jelly. In this study, we suggest that the delivery method and biophysical parameters of the umbilical cord blood as well as the pregnant age significantly affect the expression of genes from the IAP family and thus the clinical usefulness of the obtained cells.

The use of umbilical cord blood gas results to assess the condition of the newborn is crucial to evaluate their well-being after birth. Many authors agree that the threshold pH value below which fetal hypoxia is diagnosed is pH < 7.1 [16]. Scientific reports on the umbilical blood gas values in correlation with the method of delivery are ambiguous [19, 20].

The impact of the method of delivery and the pregnant age on the expression of genes from the IAP family in the mesenchymal stem cells of Wharton's jelly has not been evaluated so far.

IAP apoptosis inhibitors are a family of eight proteins (NAIP/BIRC1, cIAP1/BIRC2, cIAP2/BIRC3, XIAP/BIRC4, Survivin/BIRC5, Apollon/BIRC6, ML-AIP/BIRC7, and ILP2/BIRC8) that have the ability to regulate and inhibit the apoptosis process, inter alia through the ability to interact with caspases [21, 22]. IAPs are mainly associated with their overexpression occurring in cancer cells, which is usually linked with the resistance of tumor cells to treatment and an adverse prognosis. IAPs have multidirectional effects and a wide range of functions; besides their involvement in the pathways for programmed cell death that promotes cell survival, they are also involved in cellular differentiation, cell division, signal transduction, and cell response to damage [23–27].

The role of cIAPs in stem cells has not been explained so far. Peng et al. noted the high expression of cIAP1 protein in fetal neural progenitor cells (NPCs). The authors suggest that the cIAP1 protein plays an important role in protecting progenitor cells against TRAIL-induced apoptosis by inhibiting caspase 3 activation [28]. The function of cIAP proteins (cIAP1 and cIAP2)—promoting survival—is not limited to caspase regulation. These proteins also have the ability to activate and regulate caspase-independent pathways. It is also known that IAP family proteins have a regulating role in the mediation of signal transduction from NOD, TLR, and TNF receptors [23, 29]. They contribute to the inhibition of apoptosis through the mechanism of activating the NF*κ*B pathway in an E3-dependent manner, which promotes the expression of many molecules affecting survival, including cIAP1 and cIAP2. cIAP proteins can contribute to the activation of the NF*κ*B pathway also in the mechanism of degradation of I*κ*B inhibitors. The effect of cIAP proteins on TNF receptors (TNFR1) mediating the activation of NF*κ*B is also known. In addition, cIAPs protect cells from death by regulating the activity of receptor-interacting protein kinases 1 and 3 (RIPK) [30–32]. In addition, it is suggested that IAP proteins may participate in the Wnt signaling pathway and may also affect the activity and migration of stem cells [23]. It was also found that IAP proteins, mainly cIAP1 and cIAP2, are involved in signaling associated with innate immunity. Recent research shows that the involvement of cIAP proteins in the regulation of immune response and inflammation is based on their ubiquitin ligase activity. Ubiquitination is a process that plays an extremely important role at different levels of immune response. Such activity of IAPs allows them to regulate, among others, the NF*κ*B, MAPK, TNFR, and IRF pathways, and they are also involved in the control of inflammasome activation [30]. cIAP1 and cIAP2 proteins are important inflammasome effectors and are required for the efficient activation of caspase 1. The cIAP proteins together with the TRAF2 adapter protein interact with caspase 1, leading to its nondegenerative polyubiquitination. The TRAF2 protein interacts with the complex containing caspase-1 and is necessary for its catalytic activation and further proinflammatory effects. The deficiency of cIAP1 or cIAP2 weakens the activation of caspase 1, resulting in a suppressed inflammatory reaction in response to various agonists of the inflammasome [33].

## 2. Aim of the Study

The aim of the current work is to evaluate the expression of the *BIRC2*, *BIRC3*, and *BIRC5* genes at the level of transcription in mesenchymal stem cells derived from the umbilical cord Wharton's jelly. The study evaluated the relationship between the level of expression of the examined genes and selected biophysical parameters of the umbilical cord blood: pH, pCO_2_, pO_2_, cHCO_3_. Moreover, the relationship between the pregnant age, the method of delivery, and the level of expression of the *BIRC2*, *BIRC3*, and *BIRC5* genes was assessed.

## 3. Material

The research was carried out on stem cells isolated from umbilical cord fragments collected from 55 patients hospitalized in the Department of Obstetrics and Pathology of Pregnancy, Independent Public Clinical Hospital No. 1 in Lublin.

The recruitment criteria included healthy women in the normal course of pregnancy free from drugs, smoking, and diseases.

Sex of the newborns are as follows: female (*n* = 25) and male (*n* = 30).

Table 1 presents the characteristics of the studied group. The study was performed according to the protocol of the Bioethics Committee of the Medical University of Lublin (No. KE-0254/128/898).

## 4. Methods

Mesenchymal stem cells were isolated from the umbilical cord Wharton's jelly using the explant method [34]. To demonstrate the stem cell character of the isolated cells, cytometric analysis was performed and the presence of CD90 and CD105 surface antigens was demonstrated in the majority of the population studied [35].

To assess the phenotype of the stem cells and expression of cIAP1 and cIAP2 proteins, the following fluorescently labelled antibodies were used: PE-labeled *Mouse anti-Human* CD105 (Beckman Coulter, France); PC5-labeled *Mouse anti-Human* CD90 (Beckman Coulter, France); FITC-labeled *Rabbit anti-Human* cIAP1 (Bioss, USA); and A350-labeled *Rabbit anti-Human* cIAP2 (Bioss, USA).

Due to the fact that cIAP1 and cIAP2 proteins have intracellular localization, before proceeding with cytometric analysis, a permeation reaction of the cell membrane of the tested cells was carried out using the FIX & PERM reagent consisting of Reagent A and Reagent B (Invitrogen, Austria), according to the manufacturer's protocol. Then, the cytometric analysis was carried out according to the protocol presented in [36].

During cell culture, the fibroblast-like shape of the cells and their ability to adhere to plastic walls were confirmed. The expression of *SOX2* [35] and *POU5F1* [37] genes was demonstrated in the examined cells; also, the expression of the *SOX9* gene, characteristic of cell differentiation towards chondrocytes, was examined in cells (unpublished own study).

Cell culture and cytometric analysis were performed according to the protocol presented in our work [36].

The total cellular RNA was isolated from the obtained cells by means of the modified Chomczyński and Sacchi method [38], using TRI Reagent (Sigma, USA), chloroform (Sigma, USA), isopropanol (Sigma, USA), and ethyl alcohol (POCH, Poland). After isolation, the RNA extract was assessed using the spectrophotometric method. A reverse transcription reaction was performed on 1 *μ*g of isolated RNA according to the recommendations by the manufacturer using the High-Capacity cDNA Transcription Kits (Applied Biosystems, USA).

The expression of the *BIRC2*, *BIRC3*, and *BIRC5* genes was assessed using the qPCR method. For the study, synthesized cDNA (1 *μ*L per sample) was used, as well as reagents from Applied Biosystems: the Gene Expression Master Mix buffer and TaqMan probes (for the *BIRC2* gene: Hs_00357350_mL; for the *BIRC3* gene: Hs_00154109_mL; for the *BIRC5* gene: Hs_00153353_mL; and for the endogenous control *GAPDH*: Hs_99999905_mL [34].

The level of relative gene expression was calculated from the formula RQ = 2^−ΔΔCt^ [39]. The analysis of the expression of the studied genes was carried out using the ExpressionSuite Software v1.0.3. (Life Technologies). Statistical analysis was performed in the Statistica v13 program (StatSoft) using the Mann–Whitney *U* test and Spearman's rank correlation coefficient. Statistical significance was set at the level of *p* < 0.05.

## 5. Results

### 5.1. Cell Culture and Cytometric Analysis

**Figure 1:**
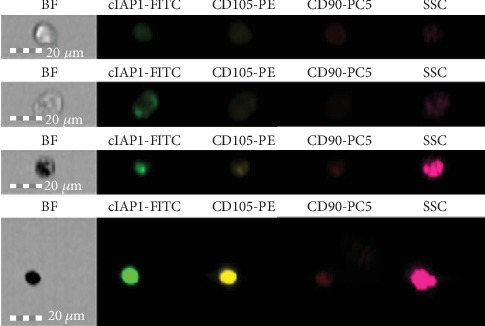
Photographs of samples mesenchymal stem cells from Wharton's jelly originated from a cell culture, presenting a bright-field microscope image and fluorescence in individual channels showing the expression of CD90 and CD105 antigens and cIAP1protein expression. The photographs were taken with the Amnis FlowSight flow cytometer. The cIAP2 expression was not visualized due to the lack of an A350 excitation UV laser in the FlowSight cytometer.

**Figure 2:**
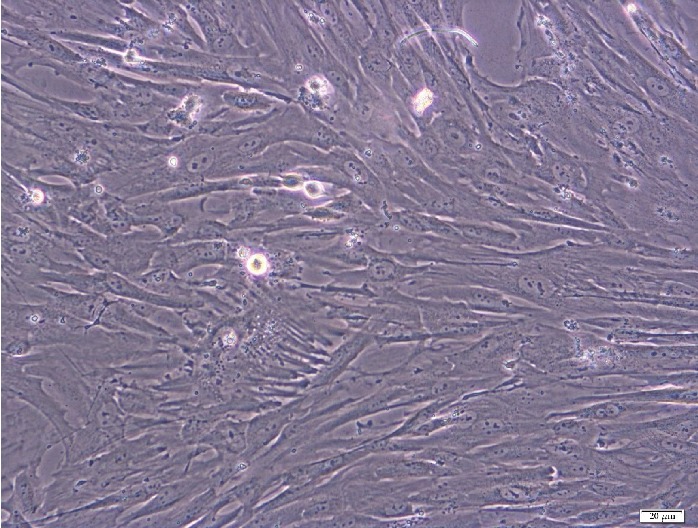
Photograph of sample stem cells from a 14-day cell culture showing a bright-field microscopic image. Photographs taken at 100x magnification using the Xcellence RT system with IX81 inverted microscope from Olympus.

The presence of the *SOX2* and *POU5F1* gene transcripts and cytometric analysis (Figure 1) as well as cell culture (Figure 2) confirmed the stem cell character of the isolated cells.

**Figure 3:**
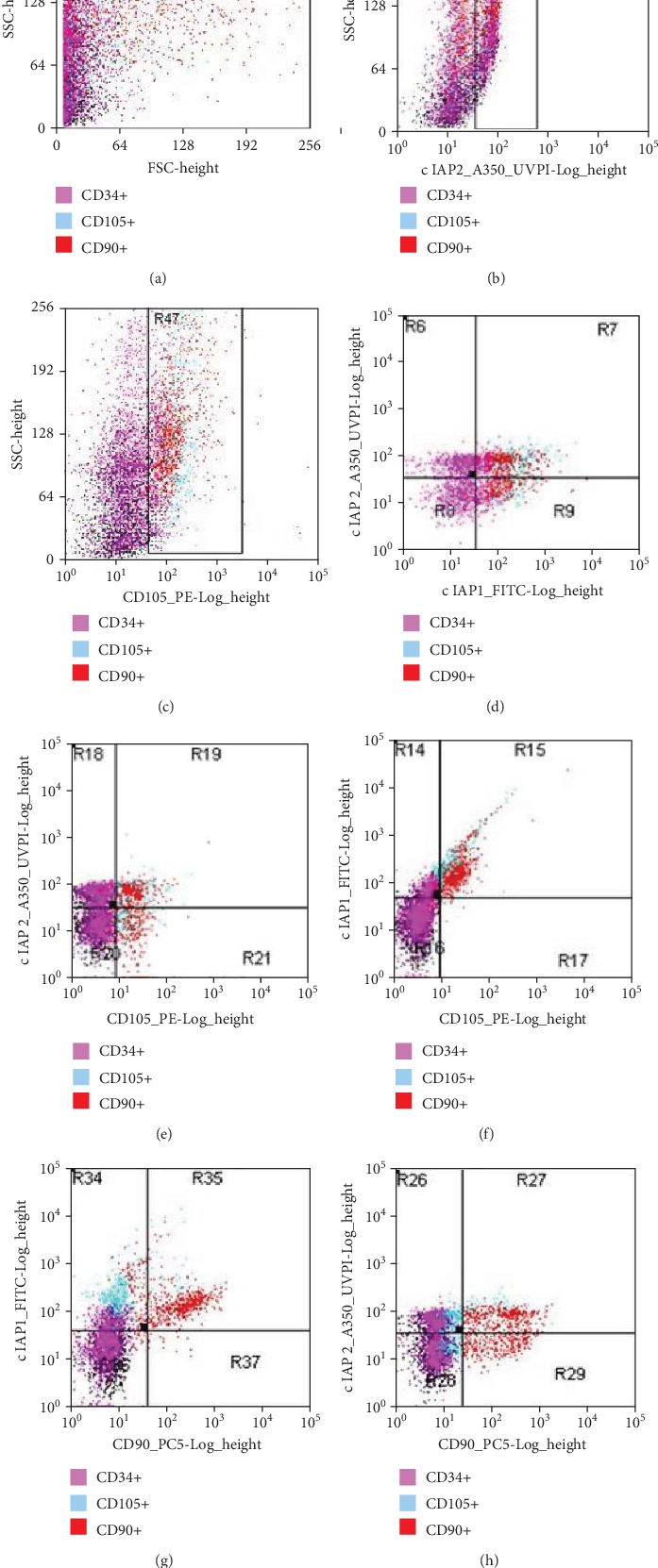
Example dot-plot graphs showing cell morphology and cell distribution in terms of expression intensity of cIAP1 and cIAP2 proteins as well as CD105 and CD90 surface antigens in a sample analyzed on cell culture day 10. (a) Dependence of the size of analyzed cells on their shape and granularity: FSC, forward scatter/SSC, side scatter. (b) cIAP2 vs. SSC: R48 cIAP2+. (c) cIAP1 vs. SSC: R47 cIAP1+. (d) cIAP1 vs. cIAP2: R6 cIAP1-/cIAP2+; R7cIAP1+/cIAP2+; R8 cIAP1-/cIAP2-; and R9 cIAP1+/cIAP2-. (e) CD105 vs. cIAP2: R18 CD105-/cIAP2+; R19 CD105+/cIAP2+; R20 CD105-/cIAP2-; and R21CD105+/cIAP2-. (f) CD105 vs. cIAP1: R14 CD105-/cIAP1+; R15CD105+/cIAP1+; R16 CD105-/cIAP1-; and R17 CD105+/cIAP1-. (g) CD90 vs. cIAP1: R34 CD90-/cIAP1+; R35 CD90+/cIAP1+; R36 CD90-/cIAP1-; and R37 CD90+/cIAP1-. (h) CD90 vs. cIAP2: R26 CD90-/cIAP2+; R27 CD90+/cIAP2+; R28 CD90-/cIAP2-; and R29 CD90+/cIAP2-. The color chart indicates the expression of the CD34+, CD90+, and CD105+ antigens, highlighting the coexpression of the surface antigens and proteins tested. Analysis and graph made using Summit™ Software digital flow cytometer together with the MoFlo XDP Beckman Coulter cell sorter.

The cytometric analysis confirmed the presence of cIPA1 and cIAP2 proteins in the analyzed cells (Figures 1 and 3). Over 50% of cells tested showed high levels of fluorescence for cIAP1 and cIAP2. There was no statistically significant correlation between the number of cIAP1+ and cIAP2+ cells and the level of expression of the *BIRC2* and *BIRC3* genes. Survivin protein analysis was not performed.

### 5.2. Gene Expression Analysis

Our study demonstrated the presence of the *BIRC2*, *BIRC3*, and *BIRC5* transcripts in all the examined mesenchymal stem cells derived from the umbilical cord Wharton's jelly (WJSC).

The relationships between the expression level of the examined genes and the pregnant age, method of delivery, selected physicochemical parameters of the umbilical cord blood, and basic parameters of the patient's blood morphology were analyzed.

**Figure 4:**
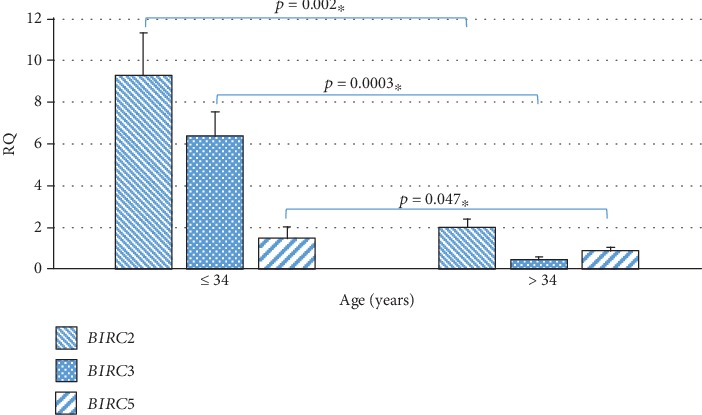
Mean expression level (RQ ± SE) of the *BIRC2*, *BIRC3*, and *BIRC5* genes in stem cells of the umbilical cord Wharton's jelly in subgroups depending on the pregnant age (≤34 years, >34 years). ^∗^The Mann–Whitney *U* test.

The analysis showed the statistically significantly higher expression of *BIRC2* (*p* = 0.002), *BIRC3* (*p* = 0.0003), and *BIRC5* (*p* = 0.047) in WJSC collected from patients up to 34 years of age compared to patients over 34 years old (Table 2, Figure 4). Analysis of the correlation between the expression level of the examined genes and the pregnant age showed statistically significant negative relationships between age and the expression of the *BIRC2* (*r* = −0.289, *p* < 0.05) and *BIRC3* (*r* = −0.318, *p* < 0.05) genes (Table 3).

**Figure 5:**
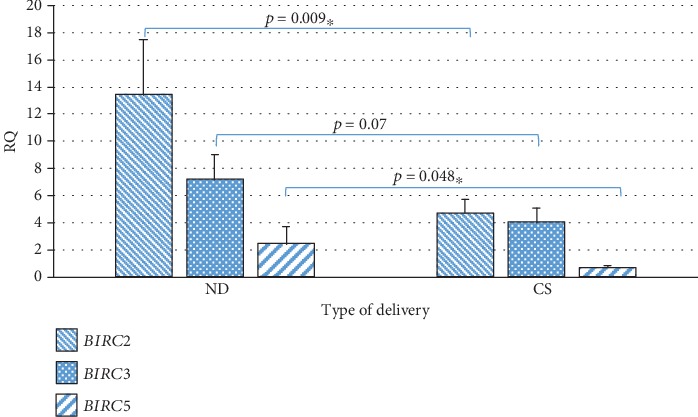
Mean expression level (RQ ± SE) of the *BIRC2*, *BIRC3*, and *BIRC5* genes in stem cells of the umbilical cord Wharton's jelly in subgroups depending on the method of delivery (ND: natural delivery, CS: cesarean section) ^∗^The Mann–Whitney *U* test.

It was demonstrated that in the group of patients giving birth naturally, the expression of the *BIRC2* (*p* = 0.009) and *BIRC5* (*p* = 0.048) genes in WJSC is statistically significantly higher, and the expression of the *BIRC3* gene (*p* = 0.07) tends to be higher in comparison with patients giving birth by cesarean section (Table 2, Figure 5).

**Figure 6:**
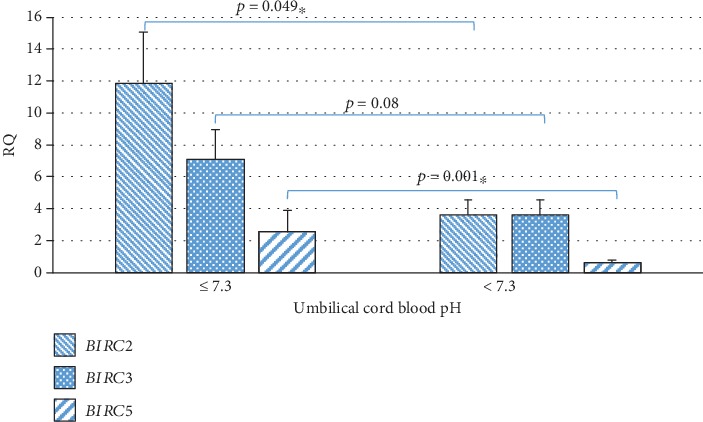
Mean expression level (RQ ± SE) of the *BIRC2*, *BIRC3*, and *BIRC5* genes in stem cells of the umbilical cord Wharton's jelly in subgroups depending on the umbilical cord blood pH (pH ≤ 7.3, pH > 7.3). ^∗^The Mann–Whitney *U* test.

The analysis of the relationship between the expression level of the examined genes and umbilical cord blood pH showed the statistically significantly higher expression the of *BIRC2* (*p* = 0.049) and *BIRC5* (*p* = 0.001) genes in WJSC and the tendency of a higher level of the *BIRC3* gene expression (*p* = 0.07) at an umbilical cord blood pH less than or equal to 7.3 compared to pH higher than 7.3 (Table 1, Figure 6). In addition, a weak negative correlation between expression level of the *BIRC2* (*r* = −0.291, *p* < 0.05) and *BIRC3* (*r* = −0.289, *p* < 0.05) genes, and the concentration of bicarbonate cHCO_3_ was demonstrated, whereas the expression level of the *BIRC5* gene (*r* = 0.325, *p* < 0.05) correlates positively with the concentration of cHCO_3_. A moderate statistically significant negative correlation was found between the expression level of the *BIRC3* gene and partial pressure of carbon dioxide (pCO_2_) (*r* = −0.525, *p* < 0.05) and between the expression level of the *BIRC5* gene and partial oxygen pressure (pO_2_) (*r* = −0.507, *p* < 0.05) (Table 3).

Statistically significant negative correlations were observed between selected parameters of the patients' blood morphology and the expression level of the examined genes; *BIRC5* negatively correlates with the number of leukocytes (WBC) (*r* = −0.507, *p* < 0.05), while *BIRC3* shows a negative relationship with platelet count (PLT) (*r* = −0.472, *p* < 0.05) (Table 3).

A statistically significant strong positive correlation was observed between the expression level of the *BIRC2* gene (*r* = 0.733, *p* < 0.05) and the expression level of the *BIRC3* gene, while the expression of the *BIRC5* gene showed a statistically significant moderate negative correlation with the expression of *BIRC2* (*r* = −0.511, *p* < 0.05) and *BIRC3* (*r* = −0.655, *p* < 0.05) (Table 3).

There were no statistically significant relationships between the expression of the *BIRC2*, *BIRC3*, and *BIRC5* genes in stem cells and the week of pregnancy in which the child was born, the number of pregnancies and deliveries, the use of oxytocin during labor, birth weight, and the sex of the infant. In the case of the sex of the infant, we observed the tendency to have over twice higher values of the *BIRC2* and *BIRC3* gene expression in stem cells in Wharton's jelly collected from women who gave birth to a son, compared with women who gave birth to a daughter. There were no differences in the *BIRC5* gene expression depending on the sex of the newborn. (Table 2).

## 6. Discussion

### 6.1. The Effect of Biophysical Parameters on the Expression of Genes Examined in WJSC

Our study evaluated the effect of the umbilical cord blood pH on the expression levels of the *BIRC2*, *BIRC3*, and *BIRC5* genes. The highest level of expression of the tested genes was obtained at pH ≤ 7.3. It was also demonstrated that the expression level of *BIRC2* is almost 3 times lower, while the expression level of *BIRC5* is over 3.5 times lower at pH > 7.3.

Studies conducted by Aufderhaar et al. showed a relationship between the concentration of hematopoietic stem cells and the course of labor, proving that a low umbilical cord blood pH and a long first stage of labor have an effect on the concentration of stem cells and their viability measured by counting the number of cell colonies [10].

Shlebak et al. also confirmed that low pH positively correlates with the number of mononuclear cells in the umbilical cord blood, and the number of CFU-GM colonies is proportional to the length of the first stage of labor [40]. In his work, Richardson et al. showed a slight drop in the umbilical cord pH during natural childbirth [19]. It can be concluded on this basis that the body's response to stress associated mainly with hypoxia occurring in the course of natural childbirth is an increase in expression compared to delivery by cesarean section.

When testing the umbilical cord blood, it was shown that the expression of the *BIRC2*, *BIRC3*, and *BIRC5* genes can be predicted basing solely on pH values. The analysis of the relationship between the expression level of the examined genes and umbilical cord blood pH showed the statistically significantly higher expression of the *BIRC2* and *BIRC5* genes in WJSC and the tendency of a higher level of the *BIRC3* gene expression at an umbilical cord blood pH less than or equal to 7.3 compared to pH higher than 7.3.

An analysis of the effect of pH on the expression of the *BIRC2*, *BIRC3*, and *BIRC5* genes demonstrated an inversely proportional correlation with selected morphology parameters. *BIRC5* correlates negatively with the number of leukocytes (WBC), while *BIRC3* shows a negative relationship with the number of platelets (PLT). Molloy et al. demonstrated an increased resistance of white blood cells to apoptosis in women after natural childbirth, stressing that leukocytes from blood collected after cesarean section do not react to liposaccharides, which indicates their reduced activity [41]. Increased resistance of mononuclear cells to apoptosis after natural childbirth demonstrated by Molloy et al. may result from the significantly higher expression of genes encoding inhibitors of apoptosis in WJSC in women giving birth naturally documented in our study.

The accumulation of reactive oxygen species can damage certain genes involved in cell growth or differentiation [42].

The obtained results confirm the hypothesis that labor contractions that generate oxidative stress have a direct impact on stem cells in the umbilical cord blood and in the umbilical cord.

### 6.2. The Effect of the Method of Delivery on the Expression of Genes Examined in WJSC

Our study also compared the method of delivery with the expression of *BIRC2*, *BIRC3*, and *BIRC5*, and statistically significant differences were observed. It was shown that in the group of patients giving birth naturally, the expression of the *BIRC2* gene is almost 3 times statistically significantly higher, the expression of the *BIRC5* gene is more than 3.5 times higher, and the expression of the *BIRC3* gene tends to have higher values in WJSC compared to patients giving birth by cesarean section. Moreover, in our previous studies, we have shown that the expression of the *POU5F1* gene, which is responsible for cell stemness, is significantly higher in Wharton's jelly stem cells from women after natural delivery, in comparison with women giving birth by caesarean section [37].

It can be suggested that the increased expression of genes examined after deliveries with longer exposure to oxidative stress (prolonged contractions) may be the result of the mobilization of stem cells from the pool of spare cells [43, 44].

At the subsequent stage, it was assumed that pCO_2_ values also have an effect on the transplantation properties of the tested material. A weak negative correlation between the expression level of *BIRC2* and the concentration of bicarbonate cHCO_3_ was demonstrated, whereas the expression level of the *BIRC5* gene correlates positively with the concentration of cHCO_3_. Moreover, a moderate statistically significant negative correlation was observed between the expression level of the *BIRC3* gene and the partial pressure of carbon dioxide (pCO_2_) and between the expression level of the *BIRC5* gene and partial oxygen pressure (pO_2_).

The studies comparing the methods of delivery were to assess differences in the umbilical cord blood gas test and red blood cell response as an exponent determining fetal well-being.

In their studies, Pomorski et al. did not obtain a significant difference in the pH of blood obtained perinatally and during the cesarean section. In earlier studies, Mancinelli et al. demonstrated a significantly higher RBC in the umbilical cord blood after natural childbirth [16].

### 6.3. The Effect of the Pregnant Age on the Expression of Genes Examined in WJSC

The conducted analysis of the relationship between the expressions of the studied genes on the gestational age did not show significant differences. However, a negative relationship between the expression of the *BIRC2* and *BIRC3* genes and the pregnant age was observed. It was demonstrated that in the group of women ≤34 years of age, the expression of the *BIRC2* gene is over 4.5 times higher and the expression of the *BIRC3* gene is more than 14 times higher, while the expression of the *BIRC5* gene is almost 2 times higher compared to patients over 34 years of age.

Research carried out by Bielec-Berek et al. demonstrated that the older the woman giving birth, the lower the average count of hematopoietic stem cells in the umbilical cord blood unit [11]. Nakagawa et al. also observed that a higher percentage of CD34+ cells in the umbilical cord blood is associated with the younger age of the mother [8].

The research carried out so far did not assess the effect of the pregnant age on the expression of the *BIRC2*, *BIRC3*, and *BIRC5* genes in mesenchymal stem cells derived from Wharton's jelly.

Our study confirms the expression of the *BIRC2*, *BIRC3*, and *BIRC5* genes in human mesenchymal stem cells, which justifies the role of IAPs in the regulation of cell survival and their protection against apoptosis. Due to the main function of IAPs, which is the inhibition of the apoptosis process, the higher expression of the *BIRC2*, *BIRC3*, and *BIRC5* genes we have demonstrated suggests that stem cells collected from younger women giving birth naturally probably are more resistant to apoptosis.

The demonstrated expression of *BIRC2* and *BIRC3* in the examined stem cells may also affect the immunomodulatory properties of stem cells, as well as their adhesion and migration ability. Literature studies suggest that IAP E3 ligase activity also has an effect on the regulation of cell shape, their migration, activity, and differentiation [23]. IAPs contribute to regulating cell adhesion and migration through their ability to activate NF*κ*B, which in turn leads to the activation of gene expression and fibronectin secretion. In addition, cIAP1 and cIAP2 are able to activate the cytoskeleton, as well as to control activation and stabilization of Rho protein, a GTPase responsible for signaling within a cell and regulation of F-actin organization [24].

Moreover, in the light of our previous research [34] in which we suggested that *BIRC5* is involved in maintaining the state of pluripotency and stem cell differentiation, the higher *BIRC5* expression demonstrated in the current study may indicate a more stem cell-like character of the isolated cells, their greater potential for differentiation, and thus better clinical utility of stem cells obtained from the umbilical cord from women after a natural childbirth.

The demonstrated strong positive relationship between the expression of *BIRC2* and *BIRC3* indicates similar functions performed by these genes in the studied material. In turn, the observed negative relationship between the expression of *BIRC2* and *BIRC3* and the expression of *BIRC5* suggests that *BIRC5*, although it belongs to the IAP family like *BIRC2* and *BIRC3*, is involved in different mechanisms in the mesenchymal stem cells of Wharton's jelly. Furthermore, it was observed that the higher the concentration of cHCO_3_, the lower the expression of *BIRC2* and *BIRC3*. However, with *BIRC5*, a reverse correlation was observed: the expression of *BIRC5* increases proportionally with the bicarbonate concentration, confirming that this gene plays a different role in WJSC as compared to *BIRC2* and *BIRC3*.

In recent years in literature, gender-specific differences in differentiation potential, expansion capacity, and secretome of mesenchymal stem cell derived from bone marrow have been reported. Sammour et al. demonstrated in vitro that female bone marrow MSCs secrete more anti-inflammatory and proangiogenic factors compared to male MSCs. In vivo, using the animal model, they also showed that female MSCs have greater anti-inflammatory and proangiogenic effects compared to male MSCs in newborns [45]. Balzano et al. in their study for the first time showed that mesenchymal stem cells derived from Wharton's jelly show differences depending on the sex of the newborn. The authors observed significant differences in gene expression of OCT4, which determines stemness and the DNA-methyltransferase epigenetic modulator gene (DNMT1) in male as compared to females [46]. In subsequent studies of this group, the authors suggest that gender may affect the potential and efficiency of cell differentiation and the autophagy process of mesenchymal stem cells of Wharton's jelly [47]. In our study, we have shown a tendency to have higher *BIRC2* and *BIRC3* gene expression values in Wharton's jelly stem cells from male newborns. Perhaps stem cells taken from Wharton's jelly from male births have a different sensitivity to apoptosis, but this requires more research.

## 7. Conclusions

Our study demonstrated that the expression of the *BIRC2*, *BIRC3*, and *BIRC5* genes in mesenchymal stem cells obtained from the umbilical cord Wharton's jelly depends on the age of the woman, the method of delivery, and physicochemical parameters of the umbilical cord blood.

It was demonstrated that WJSCs collected from younger women giving birth naturally and in the acidic environment of the umbilical cord blood are characterized by a higher expression of the *BIRC2*, *BIRC3*, and *BIRC5* genes. Taking into consideration the possible functions of the proteins encoded by the studied genes, we speculate that mesenchymal stem cells collected from the umbilical cord Wharton's jelly in younger women giving birth naturally probably show greater clinical utility and higher therapeutic potential due to their more stem cell-like character, greater potential for differentiation, greater adhesion and migration ability, and greater resistance to apoptosis.

Our research may be crucial for decisions concerning the collection, multiplication, and storing of the umbilical cord mesenchymal stem cells. But further research is needed to confirm these speculations.

## Figures and Tables

**Table 1:** Parameters characterizing the study group.

Parameter	Mean	Median	Minimum	Maximum	SD
Age (years)	30.54	30.00	19.00	46.00	5.24
Number of pregnancies	1.69	1.00	1.00	8.00	1.06
Week of pregnancy	38.58	39.00	27.00	41.00	2.16
Number of deliveries	1.53	1.00	1.00	7.00	0.89
Newborn weight (g)	3287.92	3305.00	1000.00	4740.00	587.71
pH	7.31	7.33	6.91	7.42	0.10
pCO_2_ (mmHg)	43.287	41.900	27.300	67.100	8.614
pO_2_ (mmHg)	28.29	28.10	14.60	56.00	9.22
cHCO_3_ (mmol/L)	21.73	21.75	15.80	27.80	2.58
WBC (10^9^/L)	11.77	11.09	6.53	19.22	3.29
RBC (10^12^/L)	4.03	4.11	2.74	5.31	0.46
PLT (10^9^/L)	208.40	209.00	140.00	307.00	41.38

**Table 2:** Expression levels of the *BIRC2*, *BIRC3*, and *BIRC5* genes in subgroups depending on the pregnant age, umbilical cord blood pH, and method of delivery.

Gene	Group	*N*	Mean RQ	Median	SD	SE	*p* value
Age							
*BIRC2*	≤34 years	42	9.29	4.54	13.02	2.03	0.002^∗^
	>34 years	13	2.02	1.82	1.24	0.36	
*BIRC3*	≤34 years	42	6.38	2.46	7.31	1.17	0.0003^∗^
	>34 years	13	0.45	0.27	0.40	0.12	
*BIRC5*	≤34 years	42	1.48	1.27	3.63	0.57	0.047^∗^
	>34years	13	0.87	0.96	0.57	0.16	
Umbilical cord blood pH							
*BIRC2*	≤7.3	25	11.88	5.57	15.92	3.18	0.049^∗^
	>7.3	30	3.96	2.74	3.82	0.76	
*BIRC3*	≤7.3	25	7.12	1.87	8.71	1.82	0.08
	>7.3	30	3.63	0.75	4.51	0.92	
*BIRC5*	≤7.3	25	0.65	0.44	0.64	0.11	0.001^∗^
	>7.3	30	2.54	0.37	5.45	1.32	
Type of delivery							
*BIRC2*	ND	19	13.40	6.00	17.22	4.06	0.009^∗^
	CS	36	4.68	2.80	6.28	1.06	
*BIRC3*	ND	19	7.17	2.46	7.63	1.85	0.07
	CS	36	4.00	0.73	6.35	1.11	
*BIRC5*	ND	19	2.45	0.21	5.31	1.25	0.048^∗^
	CS	36	0.69	0.40	0.69	0.12	
Sex of newborn							
*BIRC2*	F	25	5.01	2.35	5.64	1.11	0.074
	M	30	10.51	4.31	14.83	2.71	
*BIRC3*	F	25	3.67	0.71	5.77	1.15	0.085
	M	30	8.12	2.17	12.34	2.38	
*BIRC5*	F	25	1.40	0.44	3.84	0.75	0.612
	M	30	1.93	0.40	3.91	0.70	

ND: natural delivery; CS: cesarean section. ^∗^The Mann–Whitney *U* test.

**Table 3:** Correlations between the expression levels of the examined genes and the pregnant age, selected physicochemical parameters of the umbilical cord blood, and the number of WBC, PLT, and RBC in the blood of pregnant women participating in the study. ^∗^*p* < 0.05 Spearman's rank correlation.

Parameter	Age	cHCO_3_	pCO_2_	pO_2_	WBC	PLT	RBC	RQ *BIRC2*	RQ *BIRC3*	RQ *BIRC5*
RQ *BIRC2*	-0.289^∗^	-0.291^∗^	-0.314	0.136	-0.130	-0.229	-0.109		0.733^∗^	-0.511^∗^
RQ *BIRC3*	-0.318^∗^	-0.289^∗^	-0.525^∗^	0.164	-0.200	-0.472^∗^	-0.240	0.733^∗^		-0.655^∗^
RQ *BIRC5*	0.215	0.325^∗^	0.288	-0.507^∗^	-0.476^∗^	0.340	0.346	-0.511^∗^	-0.655^∗^	

## Data Availability

The data used to support the findings of this study are included within the article.
